# Efficacy of MR-guided focused ultrasound ablation for localized adenomyosis in comparison to leiomyoma

**DOI:** 10.1186/2050-5736-3-S1-O95

**Published:** 2015-06-30

**Authors:** Heidi Coy, Nelly Tan, Daniel Margolis, Peiyun Lu, Grace Kim, Matthew Brown, David Lu, Jonathan Goldin, Steven Raman

**Affiliations:** 1 University of California at Los Angeles, Los Angeles, California, United States

## Background/introduction

Symptomatic localized adenomyosis is generally treated conservatively, or with more radical treatments such as hysterectomy. An effective non-invasive therapy is needed, especially for those who wish to preserve their fertility. MR-guided Focused Ultrasound ablation (MRgFUS) has been shown as an effective treatment for symptomatic uterine leiomyomas with a large non-perfused volume (NPV) immediately after treatment. Our specific aim was to compare the change in NPV in subjects with localized adenomyomas treated with MRgFUS, with the change in NPV in subjects treated with MRgFUS for symptomatic uterine leiomyomas to determine if similar results were achieved in the localized adenomyoma cohort.

## Methods

With IRB approval, we performed a HIPAA compliant retrospective review of women treated with MRgFUS at our comprehensive treatment center for symptomatic localized adenomyosis. We matched cases with leiomyoma bearing-controls with the following criteria: 1) Similar baseline total lesion volume. 2) Similar number of lesions at baseline. 3) Similar age. Subjects who had imaging performed both prior and immediately post-treatment were included. All lesions were contoured on the T1 post-contrast sagittal series, and volumes were calculated with a validated proprietary software. All contours were confirmed by two genitourinatry trained radiologists. The software automatically calculated the total lesion volume and its perfused volume, from which we derived the NPV by subtracting the perfused lesion volume from the total lesion volume.

## Results and conclusions

10 lesions in 9 subjects were analyzed. Adenomyoma and leiomyoma cohorts were similar in baseline characteristics including age (42 *vs*. 43) and mean baseline total lesion volume (96cc *vs*. 97cc), respectively. Post-tx characteristics were also similar between adenomyoma and leiomyoma cohorts in mean total lesion volume (97cc *vs*. 90 cc), mean post-tx NPV (41cc *vs*. 52cc) and sonication number (74 *vs*. 65), respectively.

From our results we can conclude that MRgFUS can offer significant reduction in perfused volume of localized adenomyosis similar to leiomyoma, and may offer complete ablation of the adenomyoma. Therefore indicating MRgFUS may be a viable non-invasive treatment for patients with symptomatic adenomyosis, and may offer an alternative to conventional therapies.

